# Estimated greenhouse gas emissions associated with unnecessary intravenous antimicrobials administered in the hospital setting

**DOI:** 10.1017/ash.2025.10176

**Published:** 2025-10-14

**Authors:** Anil J. Chakravorty, Nicholas J. Newman, Courtney Veltri, Emily S. Spivak, Michelle T. Hecker, Leila S. Hojat

**Affiliations:** 1 Case Western Reserve University School of Medicine, Cleveland, OH, USA; 2 Department of Pharmacy, University Hospitals Cleveland Medical Center, Cleveland, OH, USA; 3 Department of Medicine, University of Utah School of Medicine, Salt Lake City, UT, USA; 4 Department of Medicine, The MetroHealth System, Cleveland, OH, USA; 5 Department of Medicine, University Hospitals Cleveland Medical Center, Cleveland, OH, USA

## Abstract

**Background:**

Healthcare has deleterious impacts on the environment through production of massive amounts of waste leading to generation of greenhouse gas (GHG) emissions. Single-use materials used for preparation and administration of intravenous (IV) medications are a large component of hospital waste. Transitioning medications from the IV-to-oral (PO) route, called switch therapy, may be a means of decreasing unnecessary waste and associated emissions arising from hospital care.

**Methods:**

This was a retrospective cohort study involving adult patients receiving IV antimicrobials with a highly bioavailable PO equivalent at a large academic medical center. For a randomly selected subset of patients, the mean number of IV days of therapy (DOT) for which PO therapy could have been administered based on our institution’s policy was determined for each antimicrobial. This proportion was applied to the full cohort to estimate the total unnecessary IV DOT. A GHG emissions estimation tool was used to estimate the emissions generated from the excess antimicrobials.

**Results:**

During the study period, 15,037 IV DOT were administered, of which 9,694 DOT (64%) were estimated to be unnecessary. This was estimated to have generated 2,049 kilograms of total waste and 0.353 metric tons of carbon dioxide equivalents, equivalent to 904 miles driven.

**Conclusions:**

Optimizing IV-to-PO antimicrobial switch policies may be an effective way to decrease hospital environmental impact through reduction of single-use supply waste and associated emissions. Future work should prioritize evaluating other potential antimicrobial stewardship interventions as a means to reduce GHG emissions.

## Introduction

Climate change and anthropogenic pollution have become increasingly prominent threats to human health at the population level. Exposure to aerosolized particulate matter is associated with increased risk of myocardial infarctions^1^ and chronic obstructive pulmonary disease exacerbations.^2^ Anthropogenic greenhouse gas (GHG) emissions catalyze unpredictable weather changes, promoting more violent natural disasters that can devastate entire populations. As sea levels rise and worldwide rates of coastal flooding increase, island nations such as the Maldives face the catastrophic possibility of submergence.^3^

Healthcare has been recognized as a major driver of the deleterious environmental impacts of human industry, accounting for approximately 8.5% of United States GHG emissions.^4^ Plastic is a major contributor to hospital waste, with some studies placing it as the number one contributor to solid waste generated by hospitals.^5,6^ Hospital-generated plastic waste must either be processed in landfills, contributing to GHG emissions and microplastic production as it is broken down, or be incinerated which releases toxic pollutants and potential carcinogens such as heavy metals, HCl, dioxins, and furans into global circulation.^7^

Within the hospital setting, intravenous (IV) bags and plastic tubing account for a large proportion of hospital plastic waste. In a study involving large healthcare facilities in Massachusetts, IV bags and tubing comprised, on average, 17% of all plastic waste generated by the facility, amounting to approximately 66 tons of plastic waste per year, illustrating the large environmental burden inherent to IV therapy.^7^

Transitioning antimicrobials with high bioavailability from the IV-to-oral (PO) route, often referred to as switch therapy, or sometimes, sequential or step down therapy,^8^ is a well-established antimicrobial stewardship practice which a large body of evidence illustrates is financially beneficial for both patient and healthcare systems while not compromising clinical success.^9^ At the healthcare system level the cost of a daily defined dose of a given antimicrobial can be two to ten-fold lower when given orally compared to parenteral delivery.^10^ In addition to lower direct drug acquisition costs, switch therapy decreases indirect antimicrobial costs including those of managing IV antimicrobial associated adverse events, laboratory drug monitoring, personnel time for preparation and administration, and length of hospitalization.^11^

Despite evidence that appropriate switch therapy is beneficial and that plastic waste produced by healthcare negatively impacts the environment, there has been little research into the impact of switch therapy on the reduction of plastic waste in hospital systems. This study involving a large academic medical center investigated how much plastic waste could be avoided if all appropriate patient candidates were transitioned from IV-to-oral (PO) antimicrobials and estimated the avoidable GHG emissions generated from the associated waste products.

## Methods

### Intervention

Our hospital system has a longstanding policy for pharmacy-initiated IV-to-PO switch therapy, which allows for conversion from the IV-to-PO route for antimicrobial and non-antimicrobial agents considered to have high bioavailability when administered enterally. The antimicrobials within the policy include azithromycin, ciprofloxacin, clindamycin, doxycycline, fluconazole, isavuconazole, letermovir, levofloxacin, linezolid, metronidazole, minocycline, rifampin, and voriconazole. Patients are ineligible for IV-to-PO conversion of antimicrobials if any of the following criteria are present: no enteral route available, hemodynamic instability requiring vasopressors, mechanical ventilation, altered mental status, active gastrointestinal suctioning or bleeding, specific indication for the IV formulation, cystic fibrosis or other known malabsorptive disorder, seizures (for antiepileptics only), or other specified exclusion to PO intake noted by the provider. Specific indications for IV antimicrobials for purposes of this analysis included IV metronidazole for *C. difficile*, IV clindamycin for necrotizing fasciitis, and IV levofloxacin for meningitis or encephalitis.

For patients with no exclusion criteria, pharmacy can discontinue the IV medication order and place a new order for the equivalent oral formulation of the drug, noting the change being performed per policy in the order comments field. Pharmacists are not required to contact the provider but are encouraged to speak with them if there are questions. Pharmacists document the encounter and its outcome in internal pharmacy communications which can be used to monitor implementation of and adherence to the policy.

### Study design and population

This was a retrospective cohort study within a large academic medical center in Cleveland, Ohio. The study was approved by the University Hospitals Institutional Review Board (STUDY20240291). We included adult patients hospitalized from October 1, 2023, to September 30, 2024, receiving IV antimicrobials listed in our pharmacy-initiated IV-to-PO conversion policy as described above. Patients were identified through extraction of medication administrations from the electronic medical record.

Of the full cohort of patients meeting these criteria, we randomly selected a cohort of up to 20 cases per antimicrobial agent included in the policy for more detailed analysis. Additional data elements extracted via chart review for the subset of patients included length of IV therapy, diet, concomitant PO medications, hospital unit type, and any exclusions from the pharmacy policy such as no enteral route or clinical instability. Hospital unit was based on the patient location at the time the antimicrobial order was placed. If exclusions were present upon treatment initiation but resolved during the IV course, we identified the date of resolution to calculate length of IV therapy after these criteria were no longer present.

### Analysis

We performed a descriptive analysis of patient characteristics, IV therapy, and switch therapy exclusions for the subset of cases which underwent chart review. For each antimicrobial agent, we determined the percentage of encounters which had no exclusions to IV-to-PO switch based on the policy criteria, that is, the percentage of encounters with an opportunity for IV-to-PO switch. For encounters in which there was an opportunity for IV-to-PO switch, we calculated the total IV days of therapy (DOT) that could have been switched to PO therapy based on the difference between the start and end dates of treatment. For those with exclusions which resolved during therapy, we calculated the difference between the exclusion resolution date and end date of treatment. If the exclusion did not resolve during the course of therapy, the case would be classified as having no potential days saved. The mean unnecessary IV DOT was calculated by dividing the total unnecessary IV DOT by the number of encounters with an opportunity for IV-to-PO switch. The percentage of encounters with an opportunity for IV-to-PO switch and the mean unnecessary days of IV therapy for each antimicrobial were then applied to the full cohort to estimate the total unnecessary days of IV antimicrobials over the one-year period.

The total annual estimated DOT saved for each antimicrobial agent was input into an antimicrobials emissions calculator tool developed to estimate the carbon dioxide equivalents associated with materials used for packaging, preparation, and administration of antimicrobials in the hospital setting, described in more detail separately.^12^ No calculator data are available for voriconazole or letermovir, thus these agents were omitted from this component of the analysis.

## Results

During the 1-year period, 3,745 patient encounters were identified in which patients received one of the antimicrobials listed in our IV-to-PO policy. A total of 207 unique cases of IV antimicrobials administered in hospitalized patients were reviewed, including 20 cases for each antimicrobial except those which had fewer than 20 administrations available to review (isavuconazole, letermovir, minocycline, rifampin) (Table 1). The majority of cases had antimicrobials initiated in an acute care ward. Most patients (*n* = 183, 88%) had an order for a PO diet and/or were concomitantly receiving other PO medications (*n* = 165, 80%). Approximately one-third of patients met at least one of the exclusion criteria, most commonly mechanical ventilation (21%) or no available enteral route or altered mental status (both 11%). Of the 76 patients with at least one exclusion to PO antimicrobials, 11 (14.5%) had resolution of the exclusion during the course of therapy and thus would have been eligible for IV-to-PO switch at that point.

**Table 1.** Characteristics for the subset of encounters reviewed (*n* = 207)

Characteristic	*n* (%)
IV Antimicrobial	207
Azithromycin	20 (9.7)
Ciprofloxacin	20 (9.7)
Clindamycin	20 (9.7)
Doxycycline	19 (9.2)
Fluconazole	20 (9.7)
Isavuconazole	11 (5.3)
Letermovir	3 (1.4)
Levofloxacin	20 (9.7)
Linezolid	20 (9.7)
Metronidazole	20 (9.7)
Minocycline	8 (3.9)
Rifampin	9 (4.3)
Voriconazole	17 (8.2)
Hospital unit type	
Acute care ward	164 (79.2)
Intensive care unit	35 (16.9)
Emergency department	8 (3.9)
PO Diet^a^	183 (88.4)
Taking other PO medications	165 (79.7)
Exclusions to PO antimicrobials^b^	76 (36.7)
Mechanically ventilated	43 (20.8)
No enteral route available	23 (11.1)
Altered mental status	22 (10.6)
Hemodynamically unstable	11 (5.3)
Infection requiring IV treatment	1 (.5)
Active GI bleed	1 (.5)
Cystic fibrosis	1 (.5)

a PO diet could include full (*n* = 109), liquid (*n* = 9), tube feeds (*n* = 52), sips with meds (*n* = 11), or other PO diet (*n* = 2).

b Sum of exclusions is greater than the total number of cases with exclusions due to some cases meeting multiple exclusion criteria. Abbreviations: PO, oral/enteral; IV, intravenous; GI, gastrointestinal.

The proportions of cases with no exclusions to PO antimicrobial treatment for each agent are listed in Table 2. The percentage of encounters with an opportunity for IV-to-PO switch for each antimicrobial ranged from as low as 40% for fluconazole to as high as 100% for letermovir.

**Table 2.** Summary data for the subset of encounters reviewed and for the full 1-year cohort

IV Antimicrobial	Subset		Full Cohort		
	Percent of total encounters with opportunity for PO switch (Total encounters reviewed)	Mean unnecessary IV DOT per encounter with opportunity for PO switch	Estimated total encounters with opportunity for PO switch (Total encounters)	Total IV DOT	Estimated total unnecessary IV DOT
Azithromycin	85 (20)	3.1	1 008 (1186)	3 380	3 125
Ciprofloxacin	80 (20)	5.1	194 (243)	1 184	991
Clindamycin	65 (20)	2.8	255 (392)	1 276	713
Doxycycline	63.2 (19)	3.4	130 (206)	867	443
Fluconazole	40 (20)	6.5	154 (386)	1 911	1 004
Isavuconazole	45.5 (11)	7.8	10 (23)	158	82
Letermovir	100 (3)	1.7	3 (3)	5	5
Levofloxacin	75 (20)	3.6	194 (258)	1 016	697
Linezolid	65.0 (20)	5.5	78 (120)	582	429
Metronidazole	60 (20)	3.9	523 (871)	4 274	2038
Minocycline	50 (8)	11	8 (15)	168	82
Rifampin	55.6 (9)	11.5	4 (7)	111	45
Voriconazole	47.1 (17)	2.4	17 (35)	105	40
Total			2 577 (3745)	15 037	9 694

Abbreviations: IV, intravenous; PO, enteral; DOT, days of therapy.

**Figure 1.**
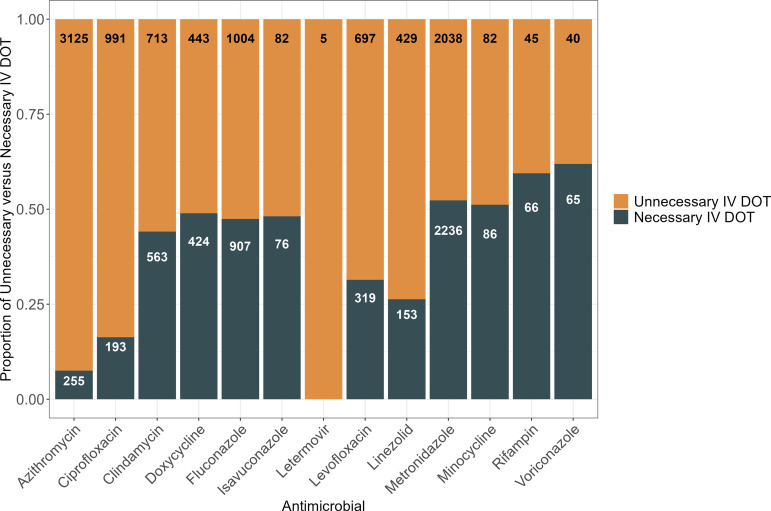
Relative proportions of estimated unnecessary versus necessary intravenous (IV) days of therapy (DOT) for each antimicrobial applied to the full cohort. Column labels refer to the total unnecessary and necessary IV DOT for each antimicrobial.

The mean unnecessary IV DOT per encounter ranged from 1.7 for letermovir to 11.5 for rifampin. When applying the data from the chart review subset of encounters to the entire cohort, of the total 15,037 IV DOT administered over one year, 9,694 (64%) were estimated to be unnecessary. Encounters in which azithromycin was administered accounted for the highest number of encounters estimated to have an opportunity for IV-to-PO switch and the highest estimated total number of unnecessary IV DOT, with an average of 3.1 unnecessary IV DOT per case in the subset of patients reviewed, equivalent to an estimated 3,125 unnecessary IV DOT per year. The total estimated excess IV DOT relative to the estimated required IV DOT for each antimicrobial is shown in Figure 1.

After inputting annual excess IV DOT for each antimicrobial into the GHG emissions calculator, it was determined that the unnecessary antimicrobials generated an estimated 2,049 kilograms of waste and 0.353 metric tons of carbon dioxide equivalents, equivalent to 904 miles driven by an average gasoline-powered passenger vehicle, 40 gallons of gasoline consumed, 389 pounds of coal burned, or the energy required to maintain 23,392 fully depleted phone batteries at full charge throughout one day. It would take approximately six trees growing for ten years to re-sequester that amount of CO_2_ out of the atmosphere according to the EPA’s publicly available GHG emissions calculator.^13^

## Discussion

This study found that 2,049 kilograms of solid waste—0.353 metric tons of CO_2_ equivalents of GHG emissions—were generated through unnecessary administration of IV antimicrobials in situations where PO equivalents were clinically appropriate. Across all antimicrobials surveyed herein there were an estimated total of 9,694 IV DOT that could have been saved over one year had appropriate switch therapy been implemented, or two-thirds of IV DOT administered. This amount of waste-associated GHG emissions was estimated to be equivalent to 904 miles driven by an average gasoline-powered passenger vehicle, approximately the distance between Boston and Atlanta, and the amount of CO_2_ which six trees could sequester over a decade.

Switch therapy has been found to be beneficial from an economic standpoint at both the systems and patient care level. Antimicrobials have been found to account for up to 20% of a hospital system’s pharmaceutical expenses, and reducing this cost through early and judicious use of switch therapy can have significant financial benefit, with studies illustrating savings in the thousands of dollars or up to 40% of total healthcare costs per patient when placed on switch therapy as well as reduced lengths of stay.^11,14–16^ Judicious use of switch therapy has been shown to be consistently beneficial in multiple areas of clinical medicine, providing lower costs and shorter LOSs without negatively impacting patient mortality.^9,17^

Moreover, extreme weather events causing nationwide IV fluid shortages have demonstrated the feasibility and benefits of switch therapy without associated negative outcomes. In response to Hurricane Helene and the subsequent national IV fluid shortage, a New Jersey based hospital system reduced their IV fluid usage by 44% through a multipronged approach including IV-to-PO antimicrobial conversion, resulting in a significant decrease in average LOS with no change in mortality rate.^18^ Additionally, a clinical decision support tool developed within the Stanford system to encourage the switch from IV-to-PO antimicrobial formulations in the setting of IV shortages was calculated to have saved the hospital almost 70 thousand dollars within two years.^19^ These studies demonstrate that hospitals can successfully adapt to reduced IV medication usage with little to no negative impact on outcome metrics and marked savings at both the system and individual level.

The healthcare industry is now recognized as a major driver of environmental degradation and anthropogenic climate change.^20^ Plastics make up a large majority of hospitals’ solid waste, and there exists a growing body of literature investigating how to curb unnecessary plastic waste at the healthcare systems level.^21,22^ Many of these studies are based on the principles of a circular economy (reduce, reuse, recycle), with an outright reduction in plastic waste being an efficacious means of assuaging the environmental impacts of the healthcare sector.^22^ There is emerging literature showing switch therapy is a viable means of significantly reducing the plastic waste generated by hospital systems, with a reduction of plastic waste that reaches into the hundreds of kilograms illustrated across multiple mid-sized American hospital systems participating in early switch therapy.^23^ While the waste generated through the production and administration of PO antimicrobials is not zero, it is inherently less than the IV route given the use of substantially fewer materials. This article serves to further expand this area of investigation and provides additional data supporting the fact that early switch therapy is an appropriate means of reducing the amount of hospital generated plastic waste and downstream GHG emissions.

Notably, our study demonstrates that the existence of an IV-to-PO switch policy alone is insufficient without a structured approach integrating clinical workflows, decision support, education, and appropriate stewardship oversight. While developing guidelines and switch therapy policies is a critical step, the meaningful benefits of these policies will only be realized with the support of antimicrobial stewardship programs which educate clinicians about the importance of prudent IV-to-PO conversion while providing them with the real world tools to facilitate this at the clinical level.

Our study has many notable strengths, one including its population and setting. This study was conducted at a large academic center and selected a sample from a diverse population within a large metropolitan area. Additionally, it is one of the first to characterize and calculate plastic waste generated by antimicrobial use while simultaneously defining new opportunities for intervention via early switch therapy. Furthermore, this study estimates the impact of a pharmacy-based intervention using real world data and utilization, illustrating that pharmacy-based interventions can be efficacious in reducing waste generated at the hospital level. Lastly, this is the first application of a previously developed CO_2_ equivalents estimation tool to real world data,^12^ exhibiting how stewardship clinicians can report emissions data alongside more traditional metrics such as DOT per patient-days to describe the impact of their interventions.

This study has several key limitations. Primarily, all the data collected herein were from one hospital which despite representing a large and heterogeneous population may limit the generalizability of the results. This review is also inherently limited by its nature as a retrospective study. While there may have been no documented contraindication to PO antimicrobials, it is impossible to know the whole scope of the subjects’ clinical picture during the time of antimicrobial administration. To combat this, we used the same rigorous and algorithmic set of standards that pharmacists at this institution use to determine if a patient is an appropriate candidate for switch therapy. In doing so these authors followed the same objective standards that would have been used in real time during the subjects’ care to determine if they were eligible for switch therapy. Another limitation is the assumption that the ratio of eligible patients for PO conversion remained consistent between the subset which underwent chart review and the full cohort. We sought to minimize this potential inconsistency by evaluating each antimicrobial agent separately, though a sample size of twenty per agent may pose a risk of over- or under-estimating the number of equivalent PO conversions at the population level. Importantly, the goal of this study was to estimate the environmental burden of IV antimicrobial use and the potential magnitude of opportunities available to stewardship programs for reducing plastic waste within a large hospital setting, which necessitated favoring large-scale approximation while limiting precision. Finally, this study did not incorporate a comprehensive environmental life cycle assessment, which in addition to the GHG emissions generated by disposal of IV antimicrobial associated waste would assess all the environmental impacts associated with raw material extraction, manufacturing, and fossil fuel consumption during transportation of these materials.

Sustainability is being investigated across all fields of medicine with studies identifying a variety of interventions that can significantly reduce the detrimental impacts of the healthcare sector specifically through reduction of waste generation, such as by implementing reusable versus single use medical equipment or isolation gowns,^24,25^ and reducing unnecessary IV medication use. Future work must continue to emphasize strategies that investigate how to impactfully decrease hospital waste across multiple sectors and improve adherence to preexisting policies such as pharmacy-directed IV-to-PO conversion. Replicating these study methods within a multi-center model would expand the observed sample size and increase the generalizability of the observed results while providing a broader assessment of the scope of the problem. Additionally, expanding this line of investigation to include non-antimicrobial IV drugs as well as the impact of PO antimicrobial and non-antimicrobial drugs is imperative to help broaden our understanding of how these treatments impact the planet and how these effects can be mitigated.

The healthcare sector is a large contributor to harmful anthropogenic impacts on the environment. Several strategic interventions can ameliorate healthcare’s impact, such as judicious switch therapy. This study found that optimizing IV-to-PO conversion can save thousands of kilograms of solid waste. These results encourage further exploration into implementing different means by which stewardship can reduce a hospital’s environmental footprint.

## Data Availability

Data used in this study may be made available upon reasonable request to the corresponding author.
